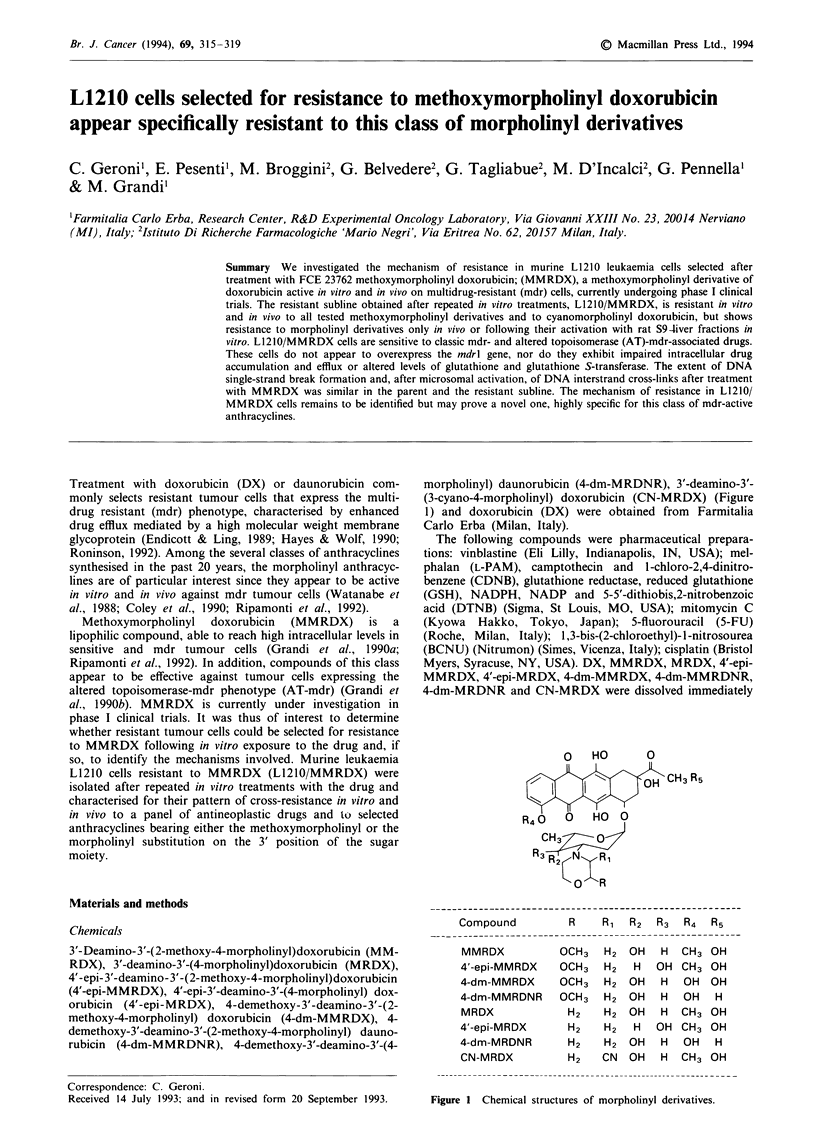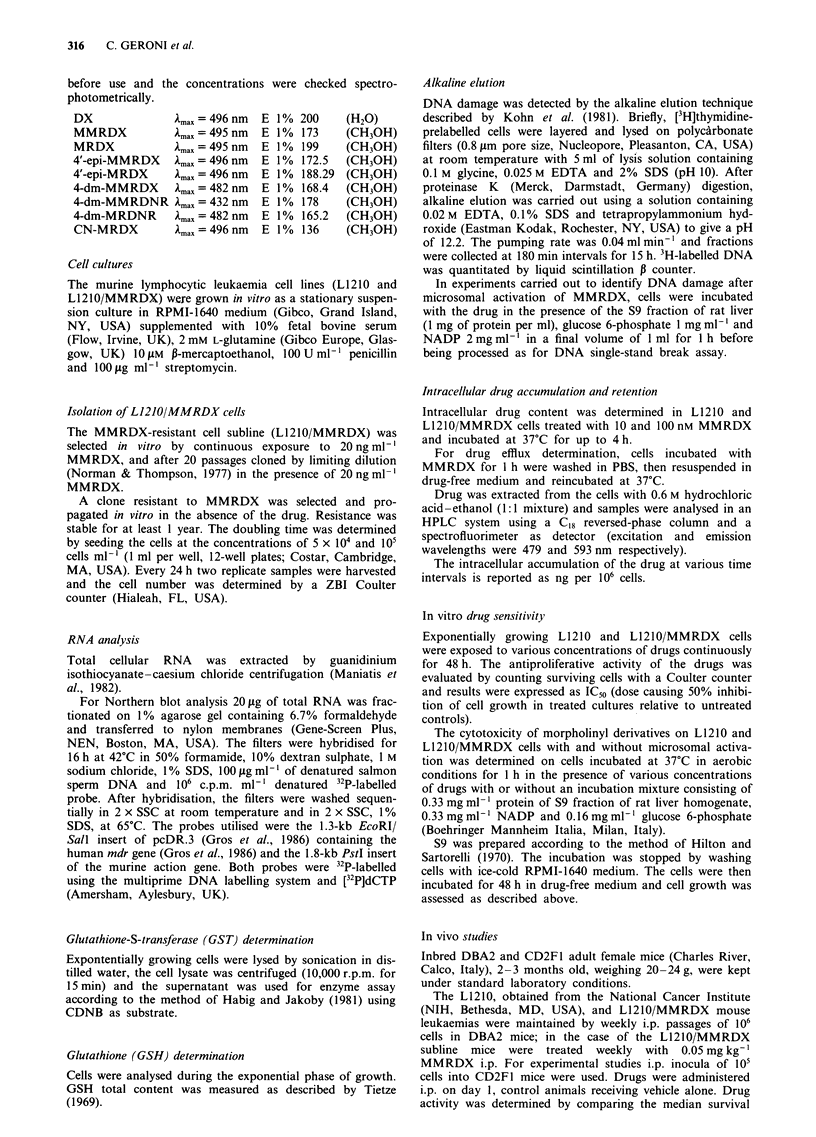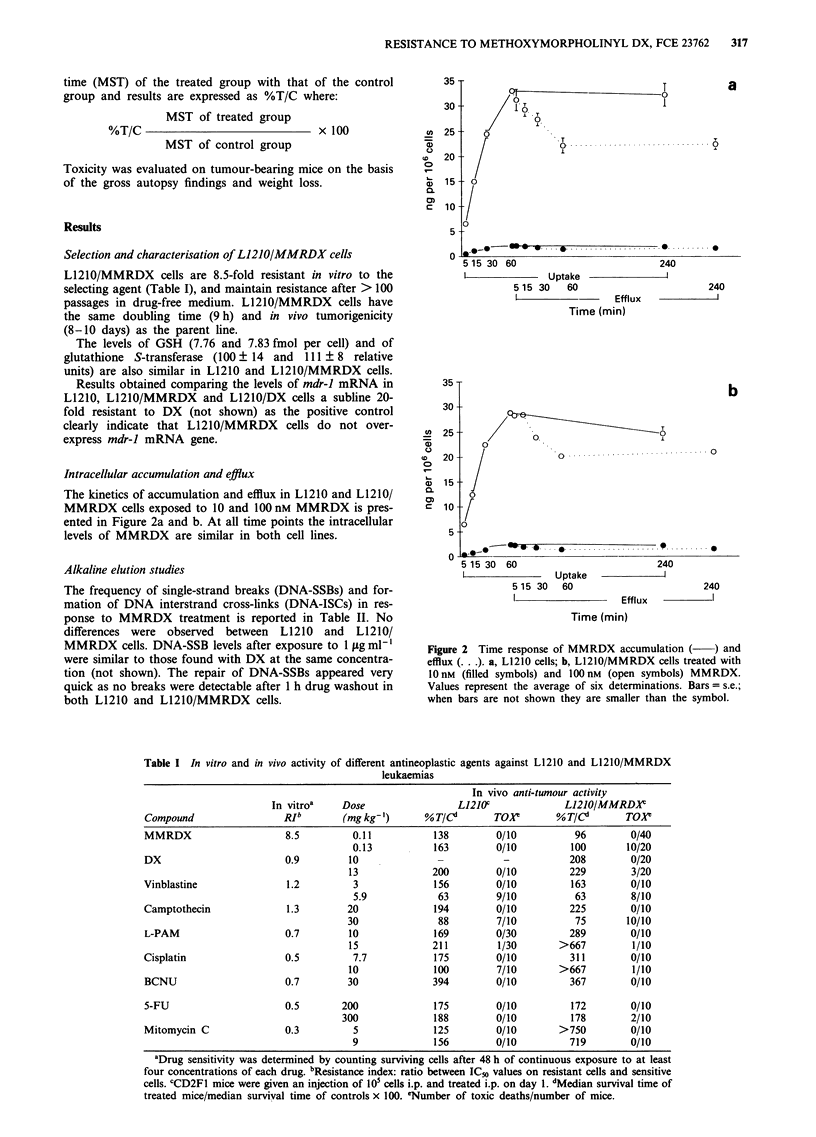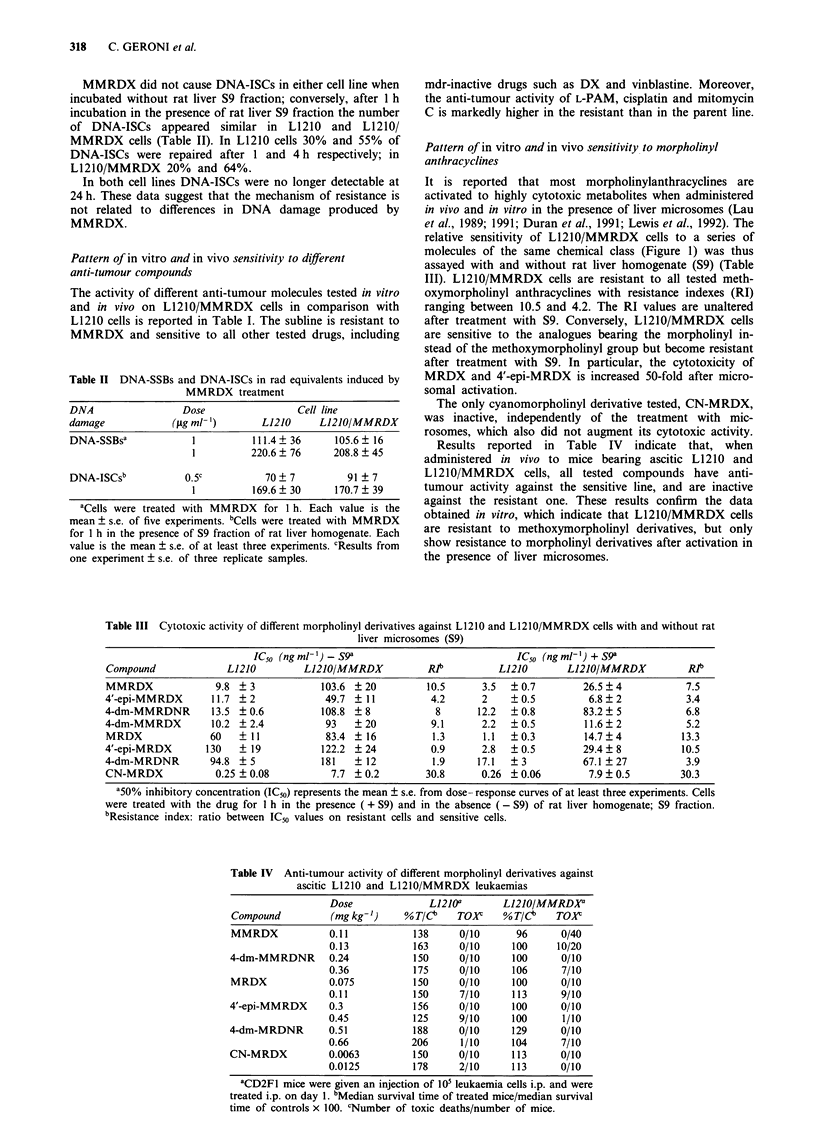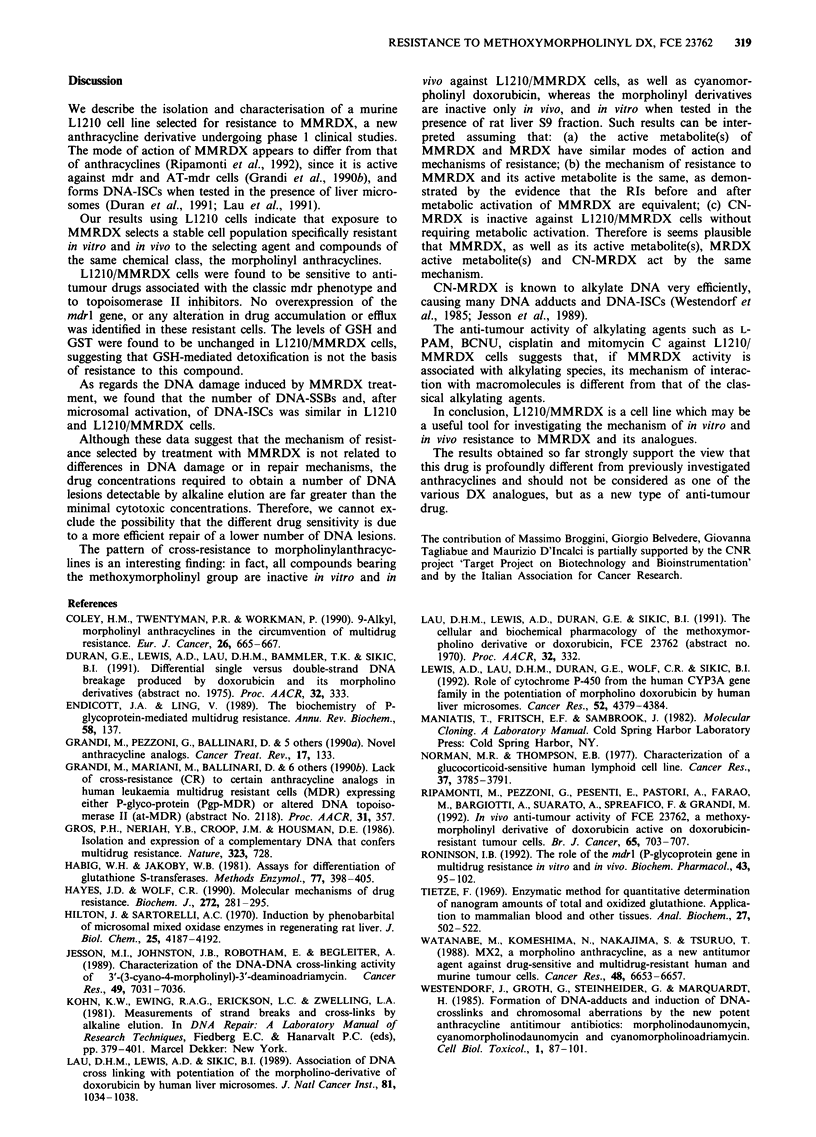# L1210 cells selected for resistance to methoxymorpholinyl doxorubicin appear specifically resistant to this class of morpholinyl derivatives.

**DOI:** 10.1038/bjc.1994.57

**Published:** 1994-02

**Authors:** C. Geroni, E. Pesenti, M. Broggini, G. Belvedere, G. Tagliabue, M. D'Incalci, G. Pennella, M. Grandi

**Affiliations:** Farmitalia Carlo Erba, Research Center, R&D Experimental Oncology Laboratory, Nerviano MI, Italy.

## Abstract

We investigated the mechanism of resistance in murine L1210 leukaemia cells selected after treatment with FCE 23762 methoxymorpholinyl doxorubicin: (MMRDX), a methoxymorpholinyl derivative of doxorubicin active in vitro and in vivo on multidrug-resistant (mdr) cells, currently undergoing phase I clinical trials. The resistant subline obtained after repeated in vitro treatments, L1210/MMRDX, is resistant in vitro and in vivo to all tested methoxymorpholinyl derivatives and to cyanomorpholinyl doxorubicin, but shows resistance to morpholinyl derivatives only in vivo or following their activation with rat S9-liver fractions in vitro. L1210/MMRDX cells are sensitive to classic mdr- and altered topoisomerase (AT)-mdr-associated drugs. These cells do not appear to overexpress the mdr1 gene, nor do they exhibit impaired intracellular drug accumulation and efflux or altered levels of glutathione and glutathione S-transferase. The extent of DNA single-strand break formation and, after microsomal activation, of DNA interstrand cross-links after treatment with MMRDX was similar in the parent and the resistant subline. The mechanism of resistance in L1210/MMRDX cells remains to be identified but may prove a novel one, highly specific for this class of mdr-active anthracyclines.


Br. J. Cancer (1994), 69, 315-319

© Macmillan Press Ltd., 1994

### L1210 cells selected for resistance to methoxymorpholinyl doxorubicin appear specifically resistant to this class of morpholinyl derivatives

C. Geroni[1], E. Pesenti[1], M. Broggini[2], G. Belvedere[2], G. Tagliabue[2], M. D'Incalci[2], G. Pennella[1] & M. Grandi[1]

[1]*Farmitalia Carlo Erba, Research Center, R&D Experimental Oncology Laboratory, Via Giovanni XXIII No. 23, 20014 Nerviano (MI), Italy;* [2]*Istituto Di Richerche Farmacologiche 'Mario Negri', Via Eritrea No. 62, 20157 Milan, Italy.*

**Summary** We investigated the mechanism of resistance in murine L1210 leukaemia cells selected after treatment with FCE 23762 methoxymorpholinyl doxorubicin; (MMRDX), a methoxymorpholinyl derivative of doxorubicin active *in vitro* and *in vivo* on multidrug-resistant (mdr) cells, currently undergoing phase I clinical trials. The resistant subline obtained after repeated *in vitro* treatments, L1210/MMRDX, is resistant *in vitro* and *in vivo* to all tested methoxymorpholinyl derivatives and to cyanomorpholinyl doxorubicin, but shows resistance to morpholinyl derivatives only *in vivo* or following their activation with rat S9-liver fractions *in vitro*. L1210/MMRDX cells are sensitive to classic mdr- and altered topoisomerase (AT)-mdr-associated drugs. These cells do not appear to overexpress the *mdr*1 gene, nor do they exhibit impaired intracellular drug accumulation and efflux or altered levels of glutathione and glutathione *S*-transferase. The extent of DNA single-strand break formation and, after microsomal activation, of DNA interstrand cross-links after treatment with MMRDX was similar in the parent and the resistant subline. The mechanism of resistance in L1210/MMRDX cells remains to be identified but may prove a novel one, highly specific for this class of mdr-active anthracyclines.

Treatment with doxorubicin (DX) or daunorubicin commonly selects resistant tumour cells that express the multidrug resistant (mdr) phenotype, characterised by enhanced drug efflux mediated by a high molecular weight membrane glycoprotein (Endicott & Ling, 1989; Hayes & Wolf, 1990; Roninson, 1992). Among the several classes of anthracyclines synthesised in the past 20 years, the morpholinyl anthracyclines are of particular interest since they appear to be active *in vitro* and *in vivo* against mdr tumour cells (Watanabe *et al*., 1988; Coley *et al*., 1990; Ripamonti *et al*., 1992).

Methoxymorpholinyl doxorubicin (MMRDX) is a lipophilic compound, able to reach high intracellular levels in sensitive and mdr tumour cells (Grandi *et al*., 1990*a*; Ripamonti *et al*., 1992). In addition, compounds of this class appear to be effective against tumour cells expressing the altered topoisomerase-mdr phenotype (AT-mdr) (Grandi *et al*., 1990*b*). MMRDX is currently under investigation in phase I clinical trials. It was thus of interest to determine whether resistant tumour cells could be selected for resistance to MMRDX following *in vitro* exposure to the drug and, if so, to identify the mechanisms involved. Murine leukaemia L1210 cells resistant to MMRDX (L1210/MMRDX) were isolated after repeated *in vitro* treatments with the drug and characterised for their pattern of cross-resistance *in vitro* and *in vivo* to a panel of antineoplastic drugs and to selected anthracyclines bearing either the methoxymorpholinyl or the morpholinyl substitution on the 3′ position of the sugar moiety.

### Materials and methods

### *Chemicals*

3′-Deamino-3′-(2-methoxy-4-morpholinyl)doxorubicin (MMRDX), 3′-deamino-3′-(4-morpholinyl)doxorubicin (MRDX), 4′-epi-3′-deamino-3′-(2-methoxy-4-morpholinyl)doxorubicin (4′-epi-MMRDX), 4′-epi-3′-deamino-3′-(4-morpholinyl) doxorubicin (4′-epi-MRDX), 4-demethoxy-3′-deamino-3′-(2-methoxy-4-morpholinyl) doxorubicin (4-dm-MMRDX), 4-demethoxy-3′-deamino-3′-(2-methoxy-4-morpholinyl) daunorubicin (4-dm-MMRDNR), 4-demethoxy-3′-deamino-3′-(4-morpholinyl) daunorubicin (4-dm-MRDNR), 3′-deamino-3′-(3-cyano-4-morpholinyl) doxorubicin (CN-MRDX) (Figure 1) and doxorubicin (DX) were obtained from Farmitalia Carlo Erba (Milan, Italy).

The following compounds were pharmaceutical preparations: vinblastine (Eli Lilly, Indianapolis, IN, USA); melphalan (L-PAM), camptothecin and 1-chloro-2,4-dinitrobenzene (CDNB), glutathione reductase, reduced glutathione (GSH), NADPH, NADP and 5-5′-dithiobis,2-nitrobenzoic acid (DTNB) (Sigma, St Louis, MO, USA); mitomycin C (Kyowa Hakko, Tokyo, Japan); 5-fluorouracil (5-FU) (Roche, Milan, Italy); 1,3-bis-(2-chloroethyl)-1-nitrosourea (BCNU) (Nitrumon) (Simes, Vicenza, Italy); cisplatin (Bristol Myers, Syracuse, NY, USA). DX, MMRDX, MRDX, 4′-epi-MMRDX, 4′-epi-MRDX, 4-dm-MMRDX, 4-dm-MMRDNR, 4-dm-MRDNR and CN-MRDX were dissolved immediately

| Compound | R | $R_1$ | $R_2$ | $R_3$ | $R_4$ | $R_5$ |
|---|---|---|---|---|---|---|
| MMRDX | $OCH_3$ | $H_2$ | OH | H | $CH_3$ | OH |
| 4′-epi-MMRDX | $OCH_3$ | $H_2$ | H | OH | $CH_3$ | OH |
| 4-dm-MMRDX | $OCH_3$ | $H_2$ | OH | H | OH | OH |
| 4-dm-MMRDNR | $OCH_3$ | $H_2$ | OH | H | OH | H |
| MRDX | $H_2$ | $H_2$ | OH | H | $CH_3$ | OH |
| 4′-epi-MRDX | $H_2$ | $H_2$ | H | OH | $CH_3$ | OH |
| 4-dm-MRDNR | $H_2$ | $H_2$ | OH | H | OH | H |
| CN-MRDX | $H_2$ | CN | OH | H | $CH_3$ | OH |

**Figure 1** Chemical structures of morpholinyl derivatives.

Correspondence: C. Geroni.

Received 14 July 1993; and in revised form 20 September 1993.

before use and the concentrations were checked spectrophotometrically.

| | | | | |
|---|---|---|---|---|
| DX | $\lambda_{max} = 496$ nm | E 1% | 200 | ($H_2O$) |
| MMRDX | $\lambda_{max} = 495$ nm | E 1% | 173 | ($CH_3OH$) |
| MRDX | $\lambda_{max} = 495$ nm | E 1% | 199 | ($CH_3OH$) |
| 4′-epi-MMRDX | $\lambda_{max} = 496$ nm | E 1% | 172.5 | ($CH_3OH$) |
| 4′-epi-MRDX | $\lambda_{max} = 496$ nm | E 1% | 188.29 | ($CH_3OH$) |
| 4-dm-MMRDX | $\lambda_{max} = 482$ nm | E 1% | 168.4 | ($CH_3OH$) |
| 4-dm-MMRDNR | $\lambda_{max} = 432$ nm | E 1% | 178 | ($CH_3OH$) |
| 4-dm-MRDNR | $\lambda_{max} = 482$ nm | E 1% | 165.2 | ($CH_3OH$) |
| CN-MRDX | $\lambda_{max} = 496$ nm | E 1% | 136 | ($CH_3OH$) |

*Cell cultures*

The murine lymphocytic leukaemia cell lines (L1210 and L1210/MMRDX) were grown *in vitro* as a stationary suspension culture in RPMI-1640 medium (Gibco, Grand Island, NY, USA) supplemented with 10% fetal bovine serum (Flow, Irvine, UK), 2 mM L-glutamine (Gibco Europe, Glasgow, UK) 10 μM β-mercaptoethanol, 100 U $ml^{-1}$ penicillin and 100 μg $ml^{-1}$ streptomycin.

*Isolation of L1210/MMRDX cells*

The MMRDX-resistant cell subline (L1210/MMRDX) was selected *in vitro* by continuous exposure to 20 ng $ml^{-1}$ MMRDX, and after 20 passages cloned by limiting dilution (Norman & Thompson, 1977) in the presence of 20 ng $ml^{-1}$ MMRDX.

A clone resistant to MMRDX was selected and propagated *in vitro* in the absence of the drug. Resistance was stable for at least 1 year. The doubling time was determined by seeding the cells at the concentrations of $5 \times 10^4$ and $10^5$ cells $ml^{-1}$ (1 ml per well, 12-well plates; Costar, Cambridge, MA, USA). Every 24 h two replicate samples were harvested and the cell number was determined by a ZBI Coulter counter (Hialeah, FL, USA).

*RNA analysis*

Total cellular RNA was extracted by guanidinium isothiocyanate–caesium chloride centrifugation (Maniatis *et al.*, 1982).

For Northern blot analysis 20 μg of total RNA was fractionated on 1% agarose gel containing 6.7% formaldehyde and transferred to nylon membranes (Gene-Screen Plus, NEN, Boston, MA, USA). The filters were hybridised for 16 h at 42°C in 50% formamide, 10% dextran sulphate, 1 M sodium chloride, 1% SDS, 100 μg $ml^{-1}$ of denatured salmon sperm DNA and $10^6$ c.p.m. $ml^{-1}$ denatured $^{32}$P-labelled probe. After hybridisation, the filters were washed sequentially in 2 × SSC at room temperature and in 2 × SSC, 1% SDS, at 65°C. The probes utilised were the 1.3-kb *Eco*RI/*Sal*I insert of pcDR.3 (Gros *et al.*, 1986) containing the human *mdr* gene (Gros *et al.*, 1986) and the 1.8-kb *Pst*I insert of the murine action gene. Both probes were $^{32}$P-labelled using the multiprime DNA labelling system and [$^{32}$P]dCTP (Amersham, Aylesbury, UK).

*Glutathione-S-transferase (GST) determination*

Expontentially growing cells were lysed by sonication in distilled water, the cell lysate was centrifuged (10,000 r.p.m. for 15 min) and the supernatant was used for enzyme assay according to the method of Habig and Jakoby (1981) using CDNB as substrate.

*Glutathione (GSH) determination*

Cells were analysed during the exponential phase of growth. GSH total content was measured as described by Tietze (1969).

*Alkaline elution*

DNA damage was detected by the alkaline elution technique described by Kohn *et al.* (1981). Briefly, [$^3$H]thymidine-prelabelled cells were layered and lysed on polycarbonate filters (0.8 μm pore size, Nucleopore, Pleasanton, CA, USA) at room temperature with 5 ml of lysis solution containing 0.1 M glycine, 0.025 M EDTA and 2% SDS (pH 10). After proteinase K (Merck, Darmstadt, Germany) digestion, alkaline elution was carried out using a solution containing 0.02 M EDTA, 0.1% SDS and tetrapropylammonium hydroxide (Eastman Kodak, Rochester, NY, USA) to give a pH of 12.2. The pumping rate was 0.04 ml $min^{-1}$ and fractions were collected at 180 min intervals for 15 h. $^3$H-labelled DNA was quantitated by liquid scintillation β counter.

In experiments carried out to identify DNA damage after microsomal activation of MMRDX, cells were incubated with the drug in the presence of the S9 fraction of rat liver (1 mg of protein per ml), glucose 6-phosphate 1 mg $ml^{-1}$ and NADP 2 mg $ml^{-1}$ in a final volume of 1 ml for 1 h before being processed as for DNA single-stand break assay.

*Intracellular drug accumulation and retention*

Intracellular drug content was determined in L1210 and L1210/MMRDX cells treated with 10 and 100 nM MMRDX and incubated at 37°C for up to 4 h.

For drug efflux determination, cells incubated with MMRDX for 1 h were washed in PBS, then resuspended in drug-free medium and reincubated at 37°C.

Drug was extracted from the cells with 0.6 M hydrochloric acid–ethanol (1:1 mixture) and samples were analysed in an HPLC system using a $C_{18}$ reversed-phase column and a spectrofluorimeter as detector (excitation and emission wavelengths were 479 and 593 nm respectively).

The intracellular accumulation of the drug at various time intervals is reported as ng per $10^6$ cells.

In vitro *drug sensitivity*

Exponentially growing L1210 and L1210/MMRDX cells were exposed to various concentrations of drugs continuously for 48 h. The antiproliferative activity of the drugs was evaluated by counting surviving cells with a Coulter counter and results were expressed as $IC_{50}$ (dose causing 50% inhibition of cell growth in treated cultures relative to untreated controls).

The cytotoxicity of morpholinyl derivatives on L1210 and L1210/MMRDX cells with and without microsomal activation was determined on cells incubated at 37°C in aerobic conditions for 1 h in the presence of various concentrations of drugs with or without an incubation mixture consisting of 0.33 mg $ml^{-1}$ protein of S9 fraction of rat liver homogenate, 0.33 mg $ml^{-1}$ NADP and 0.16 mg $ml^{-1}$ glucose 6-phosphate (Boehringer Mannheim Italia, Milan, Italy).

S9 was prepared according to the method of Hilton and Sartorelli (1970). The incubation was stopped by washing cells with ice-cold RPMI-1640 medium. The cells were then incubated for 48 h in drug-free medium and cell growth was assessed as described above.

In vivo *studies*

Inbred DBA2 and CD2F1 adult female mice (Charles River, Calco, Italy), 2–3 months old, weighing 20–24 g, were kept under standard laboratory conditions.

The L1210, obtained from the National Cancer Institute (NIH, Bethesda, MD, USA), and L1210/MMRDX mouse leukaemias were maintained by weekly i.p. passages of $10^6$ cells in DBA2 mice; in the case of the L1210/MMRDX subline mice were treated weekly with 0.05 mg $kg^{-1}$ MMRDX i.p. For experimental studies i.p. inocula of $10^5$ cells into CD2F1 mice were used. Drugs were administered i.p. on day 1, control animals receiving vehicle alone. Drug activity was determined by comparing the median survival time (MST) of the treated group with that of the control group and results are expressed as %T/C where:

$$\%T/C \; \frac{\text{MST of treated group}}{\text{MST of control group}} \times 100$$

Toxicity was evaluated on tumour-bearing mice on the basis of the gross autopsy findings and weight loss.

### Results

### *Selection and characterisation of L1210/MMRDX cells*

L1210/MMRDX cells are 8.5-fold resistant *in vitro* to the selecting agent (Table I), and maintain resistance after > 100 passages in drug-free medium. L1210/MMRDX cells have the same doubling time (9 h) and *in vivo* tumorigenicity (8–10 days) as the parent line.

The levels of GSH (7.76 and 7.83 fmol per cell) and of glutathione *S*-transferase (100 ± 14 and 111 ± 8 relative units) are also similar in L1210 and L1210/MMRDX cells.

Results obtained comparing the levels of *mdr-1* mRNA in L1210, L1210/MMRDX and L1210/DX cells a subline 20-fold resistant to DX (not shown) as the positive control clearly indicate that L1210/MMRDX cells do not over-express *mdr-1* mRNA gene.

### *Intracellular accumulation and efflux*

The kinetics of accumulation and efflux in L1210 and L1210/MMRDX cells exposed to 10 and 100 nM MMRDX is presented in Figure 2a and b. At all time points the intracellular levels of MMRDX are similar in both cell lines.

### *Alkaline elution studies*

The frequency of single-strand breaks (DNA-SSBs) and formation of DNA interstrand cross-links (DNA-ISCs) in response to MMRDX treatment is reported in Table II. No differences were observed between L1210 and L1210/MMRDX cells. DNA-SSB levels after exposure to 1 $\mu$g ml$^{-1}$ were similar to those found with DX at the same concentration (not shown). The repair of DNA-SSBs appeared very quick as no breaks were detectable after 1 h drug washout in both L1210 and L1210/MMRDX cells.

**Figure 2** Time response of MMRDX accumulation (——) and efflux (. . .). **a**, L1210 cells; **b**, L1210/MMRDX cells treated with 10 nM (filled symbols) and 100 nM (open symbols) MMRDX. Values represent the average of six determinations. Bars = s.e.; when bars are not shown they are smaller than the symbol.

**Table I** *In vitro* and *in vivo* activity of different antineoplastic agents against L1210 and L1210/MMRDX leukaemias

| | | | In vivo *anti-tumour activity* | | | |
|---|---|---|---|---|---|---|
| | In vitro[a] | *Dose* | *L1210*[c] | | *L1210/MMRDX*[c] | |
| *Compound* | *RI*[b] | *(mg kg$^{-1}$)* | *%T/C*[d] | *TOX*[e] | *%T/C*[d] | *TOX*[e] |
| MMRDX | 8.5 | 0.11 | 138 | 0/10 | 96 | 0/40 |
| | | 0.13 | 163 | 0/10 | 100 | 10/20 |
| DX | 0.9 | 10 | – | – | 208 | 0/20 |
| | | 13 | 200 | 0/10 | 229 | 3/20 |
| Vinblastine | 1.2 | 3 | 156 | 0/10 | 163 | 0/10 |
| | | 5.9 | 63 | 9/10 | 63 | 8/10 |
| Camptothecin | 1.3 | 20 | 194 | 0/10 | 225 | 0/10 |
| | | 30 | 88 | 7/10 | 75 | 10/10 |
| L-PAM | 0.7 | 10 | 169 | 0/30 | 289 | 0/10 |
| | | 15 | 211 | 1/30 | >667 | 1/10 |
| Cisplatin | 0.5 | 7.7 | 175 | 0/10 | 311 | 0/10 |
| | | 10 | 100 | 7/10 | >667 | 1/10 |
| BCNU | 0.7 | 30 | 394 | 0/10 | 367 | 0/10 |
| 5-FU | 0.5 | 200 | 175 | 0/10 | 172 | 0/10 |
| | | 300 | 188 | 0/10 | 178 | 2/10 |
| Mitomycin C | 0.3 | 5 | 125 | 0/10 | >750 | 0/10 |
| | | 9 | 156 | 0/10 | 719 | 0/10 |

[a]Drug sensitivity was determined by counting surviving cells after 48 h of continuous exposure to at least four concentrations of each drug. [b]Resistance index: ratio between $IC_{50}$ values on resistant cells and sensitive cells. [c]CD2F1 mice were given an injection of $10^5$ cells i.p. and treated i.p. on day 1. [d]Median survival time of treated mice/median survival time of controls × 100. [e]Number of toxic deaths/number of mice.

MMRDX did not cause DNA-ISCs in either cell line when incubated without rat liver S9 fraction; conversely, after 1 h incubation in the presence of rat liver S9 fraction the number of DNA-ISCs appeared similar in L1210 and L1210/MMRDX cells (Table II). In L1210 cells 30% and 55% of DNA-ISCs were repaired after 1 and 4 h respectively; in L1210/MMRDX 20% and 64%.

In both cell lines DNA-ISCs were no longer detectable at 24 h. These data suggest that the mechanism of resistance is not related to differences in DNA damage produced by MMRDX.

### *Pattern of* in vitro *and* in vivo *sensitivity to different anti-tumour compounds*

The activity of different anti-tumour molecules tested *in vitro* and *in vivo* on L1210/MMRDX cells in comparison with L1210 cells is reported in Table I. The subline is resistant to MMRDX and sensitive to all other tested drugs, including mdr-inactive drugs such as DX and vinblastine. Moreover, the anti-tumour activity of L-PAM, cisplatin and mitomycin C is markedly higher in the resistant than in the parent line.

**Table II** DNA-SSBs and DNA-ISCs in rad equivalents induced by MMRDX treatment

| *DNA damage* | *Dose* ($\mu g\ ml^{-1}$) | *Cell line* *L1210* | *L1210/MMRDX* |
|---|---|---|---|
| DNA-SSBs[a] | 1 | 111.4 ± 36 | 105.6 ± 16 |
| | 1 | 220.6 ± 76 | 208.8 ± 45 |
| DNA-ISCs[b] | 0.5[c] | 70 ± 7 | 91 ± 7 |
| | 1 | 169.6 ± 30 | 170.7 ± 39 |

[a]Cells were treated with MMRDX for 1 h. Each value is the mean ± s.e. of five experiments. [b]Cells were treated with MMRDX for 1 h in the presence of S9 fraction of rat liver homogenate. Each value is the mean ± s.e. of at least three experiments. [c]Results from one experiment ± s.e. of three replicate samples.

### *Pattern of* in vitro *and* in vivo *sensitivity to morpholinyl anthracyclines*

It is reported that most morpholinylanthracyclines are activated to highly cytotoxic metabolites when administered *in vivo* and *in vitro* in the presence of liver microsomes (Lau *et al.*, 1989; 1991; Duran *et al.*, 1991; Lewis *et al.*, 1992). The relative sensitivity of L1210/MMRDX cells to a series of molecules of the same chemical class (Figure 1) was thus assayed with and without rat liver homogenate (S9) (Table III). L1210/MMRDX cells are resistant to all tested methoxymorpholinyl anthracyclines with resistance indexes (RI) ranging between 10.5 and 4.2. The RI values are unaltered after treatment with S9. Conversely, L1210/MMRDX cells are sensitive to the analogues bearing the morpholinyl instead of the methoxymorpholinyl group but become resistant after treatment with S9. In particular, the cytotoxicity of MRDX and 4'-epi-MRDX is increased 50-fold after microsomal activation.

The only cyanomorpholinyl derivative tested, CN-MRDX, was inactive, independently of the treatment with microsomes, which also did not augment its cytotoxic activity.

Results reported in Table IV indicate that, when administered *in vivo* to mice bearing ascitic L1210 and L1210/MMRDX cells, all tested compounds have anti-tumour activity against the sensitive line, and are inactive against the resistant one. These results confirm the data obtained *in vitro*, which indicate that L1210/MMRDX cells are resistant to methoxymorpholinyl derivatives, but only show resistance to morpholinyl derivatives after activation in the presence of liver microsomes.

**Table III** Cytotoxic activity of different morpholinyl derivatives against L1210 and L1210/MMRDX cells with and without rat liver microsomes (S9)

| *Compound* | *$IC_{50}$ ($ng\ ml^{-1}$) − S9[a]* *L1210* | *L1210/MMRDX* | *RI[b]* | *$IC_{50}$ ($ng\ ml^{-1}$) + S9[a]* *L1210* | *L1210/MMRDX* | *RI[b]* |
|---|---|---|---|---|---|---|
| MMRDX | 9.8 ± 3 | 103.6 ± 20 | 10.5 | 3.5 ± 0.7 | 26.5 ± 4 | 7.5 |
| 4'-epi-MMRDX | 11.7 ± 2 | 49.7 ± 11 | 4.2 | 2 ± 0.5 | 6.8 ± 2 | 3.4 |
| 4-dm-MMRDNR | 13.5 ± 0.6 | 108.8 ± 8 | 8 | 12.2 ± 0.8 | 83.2 ± 5 | 6.8 |
| 4-dm-MMRDX | 10.2 ± 2.4 | 93 ± 20 | 9.1 | 2.2 ± 0.5 | 11.6 ± 2 | 5.2 |
| MRDX | 60 ± 11 | 83.4 ± 16 | 1.3 | 1.1 ± 0.3 | 14.7 ± 4 | 13.3 |
| 4'-epi-MRDX | 130 ± 19 | 122.2 ± 24 | 0.9 | 2.8 ± 0.5 | 29.4 ± 8 | 10.5 |
| 4-dm-MRDNR | 94.8 ± 5 | 181 ± 12 | 1.9 | 17.1 ± 3 | 67.1 ± 27 | 3.9 |
| CN-MRDX | 0.25 ± 0.08 | 7.7 ± 0.2 | 30.8 | 0.26 ± 0.06 | 7.9 ± 0.5 | 30.3 |

[a]50% inhibitory concentration ($IC_{50}$) represents the mean ± s.e. from dose-response curves of at least three experiments. Cells were treated with the drug for 1 h in the presence ( + S9) and in the absence ( − S9) of rat liver homogenate; S9 fraction. [b]Resistance index: ratio between $IC_{50}$ values on resistant cells and sensitive cells.

**Table IV** Anti-tumour activity of different morpholinyl derivatives against ascitic L1210 and L1210/MMRDX leukaemias

| *Compound* | *Dose* ($mg\ kg^{-1}$) | *L1210[a]* *%T/C[b]* | *TOX[c]* | *L1210/MMRDX[a]* *%T/C[b]* | *TOX[c]* |
|---|---|---|---|---|---|
| MMRDX | 0.11 | 138 | 0/10 | 96 | 0/40 |
| | 0.13 | 163 | 0/10 | 100 | 10/20 |
| 4-dm-MMRDNR | 0.24 | 150 | 0/10 | 100 | 0/10 |
| | 0.36 | 175 | 0/10 | 106 | 7/10 |
| MRDX | 0.075 | 150 | 0/10 | 100 | 0/10 |
| | 0.11 | 150 | 7/10 | 113 | 9/10 |
| 4'-epi-MMRDX | 0.3 | 156 | 0/10 | 100 | 0/10 |
| | 0.45 | 125 | 9/10 | 100 | 1/10 |
| 4-dm-MRDNR | 0.51 | 188 | 0/10 | 129 | 0/10 |
| | 0.66 | 206 | 1/10 | 104 | 7/10 |
| CN-MRDX | 0.0063 | 150 | 0/10 | 113 | 0/10 |
| | 0.0125 | 178 | 2/10 | 113 | 0/10 |

[a]CD2F1 mice were given an injection of $10^5$ leukaemia cells i.p. and were treated i.p. on day 1. [b]Median survival time of treated mice/median survival time of controls × 100. [c]Number of toxic deaths/number of mice.

### Discussion

We describe the isolation and characterisation of a murine L1210 cell line selected for resistance to MMRDX, a new anthracycline derivative undergoing phase 1 clinical studies. The mode of action of MMRDX appears to differ from that of anthracyclines (Ripamonti *et al.*, 1992), since it is active against mdr and AT-mdr cells (Grandi *et al.*, 1990*b*), and forms DNA-ISCs when tested in the presence of liver microsomes (Duran *et al.*, 1991; Lau *et al.*, 1991).

Our results using L1210 cells indicate that exposure to MMRDX selects a stable cell population specifically resistant *in vitro* and *in vivo* to the selecting agent and compounds of the same chemical class, the morpholinyl anthracyclines.

L1210/MMRDX cells were found to be sensitive to antitumour drugs associated with the classic mdr phenotype and to topoisomerase II inhibitors. No overexpression of the *mdr*1 gene, or any alteration in drug accumulation or efflux was identified in these resistant cells. The levels of GSH and GST were found to be unchanged in L1210/MMRDX cells, suggesting that GSH-mediated detoxification is not the basis of resistance to this compound.

As regards the DNA damage induced by MMRDX treatment, we found that the number of DNA-SSBs and, after microsomal activation, of DNA-ISCs was similar in L1210 and L1210/MMRDX cells.

Although these data suggest that the mechanism of resistance selected by treatment with MMRDX is not related to differences in DNA damage or in repair mechanisms, the drug concentrations required to obtain a number of DNA lesions detectable by alkaline elution are far greater than the minimal cytotoxic concentrations. Therefore, we cannot exclude the possibility that the different drug sensitivity is due to a more efficient repair of a lower number of DNA lesions.

The pattern of cross-resistance to morpholinylanthracyclines is an interesting finding: in fact, all compounds bearing the methoxymorpholinyl group are inactive *in vitro* and *in vivo* against L1210/MMRDX cells, as well as cyanomorpholinyl doxorubicin, whereas the morpholinyl derivatives are inactive only *in vivo*, and *in vitro* when tested in the presence of rat liver S9 fraction. Such results can be interpreted assuming that: (a) the active metabolite(s) of MMRDX and MRDX have similar modes of action and mechanisms of resistance; (b) the mechanism of resistance to MMRDX and its active metabolite is the same, as demonstrated by the evidence that the RIs before and after metabolic activation of MMRDX are equivalent; (c) CN-MRDX is inactive against L1210/MMRDX cells without requiring metabolic activation. Therefore is seems plausible that MMRDX, as well as its active metabolite(s), MRDX active metabolite(s) and CN-MRDX act by the same mechanism.

CN-MRDX is known to alkylate DNA very efficiently, causing many DNA adducts and DNA-ISCs (Westendorf *et al.*, 1985; Jesson *et al.*, 1989).

The anti-tumour activity of alkylating agents such as L-PAM, BCNU, cisplatin and mitomycin C against L1210/MMRDX cells suggests that, if MMRDX activity is associated with alkylating species, its mechanism of interaction with macromolecules is different from that of the classical alkylating agents.

In conclusion, L1210/MMRDX is a cell line which may be a useful tool for investigating the mechanism of *in vitro* and *in vivo* resistance to MMRDX and its analogues.

The results obtained so far strongly support the view that this drug is profoundly different from previously investigated anthracyclines and should not be considered as one of the various DX analogues, but as a new type of anti-tumour drug.

The contribution of Massimo Broggini, Giorgio Belvedere, Giovanna Tagliabue and Maurizio D'Incalci is partially supported by the CNR project 'Target Project on Biotechnology and Bioinstrumentation' and by the Italian Association for Cancer Research.

### References

COLEY, H.M., TWENTYMAN, P.R. & WORKMAN, P. (1990). 9-Alkyl, morpholinyl anthracyclines in the circumvention of multidrug resistance. *Eur. J. Cancer*, **26**, 665–667.

DURAN, G.E., LEWIS, A.D., LAU, D.H.M., BAMMLER, T.K. & SIKIC, B.I. (1991). Differential single versus double-strand DNA breakage produced by doxorubicin and its morpholino derivatives (abstract no. 1975). *Proc. AACR*, **32**, 333.

ENDICOTT, J.A. & LING, V. (1989). The biochemistry of P-glycoprotein-mediated multidrug resistance. *Annu. Rev. Biochem.*, **58**, 137.

GRANDI, M., PEZZONI, G., BALLINARI, D. & 5 others (1990*a*). Novel anthracycline analogs. *Cancer Treat. Rev.*, **17**, 133.

GRANDI, M., MARIANI, M., BALLINARI, D. & 6 others (1990*b*). Lack of cross-resistance (CR) to certain anthracycline analogs in human leukaemia multidrug resistant cells (MDR) expressing either P-glyco-protein (Pgp-MDR) or altered DNA topoisomerase II (at-MDR) (abstract No. 2118). *Proc. AACR*, **31**, 357.

GROS, P.H., NERIAH, Y.B., CROOP, J.M. & HOUSMAN, D.E. (1986). Isolation and expression of a complementary DNA that confers multidrug resistance. *Nature*, **323**, 728.

HABIG, W.H. & JAKOBY, W.B. (1981). Assays for differentiation of glutathione S-transferases. *Methods Enzymol.*, **77**, 398–405.

HAYES, J.D. & WOLF, C.R. (1990). Molecular mechanisms of drug resistance. *Biochem. J.*, **272**, 281–295.

HILTON, J. & SARTORELLI, A.C. (1970). Induction by phenobarbital of microsomal mixed oxidase enzymes in regenerating rat liver. *J. Biol. Chem.*, **25**, 4187–4192.

JESSON, M.I., JOHNSTON, J.B., ROBOTHAM, E. & BEGLEITER, A. (1989). Characterization of the DNA-DNA cross-linking activity of 3′-(3-cyano-4-morpholinyl)-3′-deaminoadriamycin. *Cancer Res.*, **49**, 7031–7036.

KOHN, K.W., EWING, R.A.G., ERICKSON, L.C. & ZWELLING, L.A. (1981). Measurements of strand breaks and cross-links by alkaline elution. In *DNA Repair: A Laboratory Manual of Research Techniques*, Fiedberg E.C. & Hanarvalt P.C. (eds), pp. 379–401. Marcel Dekker: New York.

LAU, D.H.M., LEWIS, A.D. & SIKIC, B.I. (1989). Association of DNA cross linking with potentiation of the morpholino-derivative of doxorubicin by human liver microsomes. *J. Natl Cancer Inst.*, **81**, 1034–1038.

LAU, D.H.M., LEWIS, A.D., DURAN, G.E. & SIKIC, B.I. (1991). The cellular and biochemical pharmacology of the methoxymorpholino derivative or doxorubicin, FCE 23762 (abstract no. 1970). *Proc. AACR*, **32**, 332.

LEWIS, A.D., LAU, D.H.M., DURAN, G.E., WOLF, C.R. & SIKIC, B.I. (1992). Role of cytochrome P-450 from the human CYP3A gene family in the potentiation of morpholino doxorubicin by human liver microsomes. *Cancer Res.*, **52**, 4379–4384.

MANIATIS, T., FRITSCH, E.F. & SAMBROOK, J. (1982). *Molecular Cloning. A Laboratory Manual.* Cold Spring Harbor Laboratory Press: Cold Spring Harbor, NY.

NORMAN, M.R. & THOMPSON, E.B. (1977). Characterization of a glucocorticoid-sensitive human lymphoid cell line. *Cancer Res.*, **37**, 3785–3791.

RIPAMONTI, M., PEZZONI, G., PESENTI, E., PASTORI, A., FARAO, M., BARGIOTTI, A., SUARATO, A., SPREAFICO, F. & GRANDI, M. (1992). *In vivo* anti-tumour activity of FCE 23762, a methoxymorpholinyl derivative of doxorubicin active on doxorubicin-resistant tumour cells. *Br. J. Cancer*, **65**, 703–707.

RONINSON, I.B. (1992). The role of the *mdr*1 (P-glycoprotein gene in multidrug resistance *in vitro* and *in vivo*. *Biochem. Pharmacol.*, **43**, 95–102.

TIETZE, F. (1969). Enzymatic method for quantitative determination of nanogram amounts of total and oxidized glutathione. Application to mammalian blood and other tissues. *Anal. Biochem.*, **27**, 502–522.

WATANABE, M., KOMESHIMA, N., NAKAJIMA, S. & TSURUO, T. (1988). MX2, a morpholino anthracycline, as a new antitumor agent against drug-sensitive and multidrug-resistant human and murine tumour cells. *Cancer Res.*, **48**, 6653–6657.

WESTENDORF, J., GROTH, G., STEINHEIDER, G. & MARQUARDT, H. (1985). Formation of DNA-adducts and induction of DNA-crosslinks and chromosomal aberrations by the new potent anthracycline antitimour antibiotics: morpholinodaunomycin, cyanomorpholinodaunomycin and cyanomorpholinoadriamycin. *Cell Biol. Toxicol.*, **1**, 87–101.